# Alice in Wonderland Syndrome as a Presenting Manifestation of Creutzfeldt-Jakob Disease

**DOI:** 10.3389/fneur.2019.00473

**Published:** 2019-05-09

**Authors:** Tirza Naarden, Bastiaan C. ter Meulen, Sarah I. van der Weele, Jan Dirk Blom

**Affiliations:** ^1^Department of Neurology, Onze Lieve Vrouwe Gasthuis, Amsterdam, Netherlands; ^2^Department of Neurology, Zaans Medisch Centrum, Zaandam, Netherlands; ^3^Outpatient Clinic for Uncommon Psychiatric Syndromes, Parnassia Psychiatric Institute, The Hague, Netherlands; ^4^Faculty of Social and Behavioural Sciences, Leiden University, Leiden, Netherlands; ^5^Department of Psychiatry, University of Groningen, Groningen, Netherlands

**Keywords:** etiology, Heidenhain variant, metamorphopsia, neurodegeneration, prion disease, prognosis, time distortion, visual perception

## Abstract

**Background:** Alice in Wonderland syndrome (AIWS) is a rare neurological disorder characterized by distortions of visual perception (metamorphopsias), the body image, and the experience of time, along with derealization and depersonalization. Some 85% of patients present with perceptual distortions in a single sensory modality, e.g., only visual or only somesthetic in nature. Moreover, the majority experience only a single type of distortion, e.g., only micropsia or only macropsia. AIWS has many different etiologies, and hence an extensive differential diagnosis. Its amenability to treatment depends on the underlying pathological process, which in children is mostly encephalitis, and in adults, migraine. In the literature, no more than 180 “clinical” cases of AIWS have been described (i.e., cases in need of medical attention). Of them, some 50% showed a favorable prognosis. However, non-clinical cases (i.e., fleeting, transient cases of AIWS for which no professional help is needed) have been described in up to 30% of the general population. This indicates that AIWS is perhaps not as rare as traditionally assumed, and has led some authors to conclude that, prognostically, AIWS is usually harmless.

**Methods:** From our own clinical practice, we describe the first known case of Creutzfeldt-Jakob Disease (CJD, Heidenhain variant) that presented with symptoms of AIWS.

**Results:** In our patient, disease onset was sudden and rapidly progressive, starting with isolated visual symptoms. Symptoms of AIWS comprised akinetopsia, chloropsia, micropsia, macropsia, zoom vision, and time distortions (quick-motion phenomenon and protracted duration). Soon, these were complicated by paraesthesias, gait instability, aphasia, expressive amusia, cognitive decline, and behavioral changes in the form of agitation and emotional lability. The diagnosis of probable sporadic CJD was confirmed with the aid of a head MRI and cerebrospinal fluid (14-3-3 protein). In the absence of any treatment options, our patient was discharged home and died within 2 months after his visual symptoms had begun. Autopsy consent was not obtained.

**Conclusion:** We conclude that AIWS is not always as harmless as sometimes suggested, and that CJD, although extremely rare, must be part of its extensive differential diagnosis, notably in the presence of rapid cognitive decline.

## Background

Alice in Wonderland syndrome (AIWS) is a rare neurological disorder characterized by distortions of visual perception (metamorphopsias), the body image, and the experience of time. As noted as early as 1955 by John Todd, these symptoms may be accompanied by derealization and depersonalization (1). Patients suffering from AIWS may end up consulting a neurologist or a psychiatrist, although in both specialties it is not as well-known as it deserves to be. This is at least partly due to the fact that major classifications such as the *Diagnostic and Statistical Manual of Mental Disorders* [DSM; (2)] do not list it as a diagnostic category, while others, such as the *International Classification of Diseases* [ICD; (3)] pay only limited attention to it. Another reason may be the relatively small number of published cases. A review of the extant literature, published in 2016, indicated that only 169 cases of AIWS had been described since the syndrome's conceptualization in 1955, which boils down to a mean number of 1.1 cases per year (4). Although this number has been rising steadily over the past few years, it currently lies around 180. Of note, these are all cases in need of medical attention, i.e., what we call “clinical cases.” Meanwhile, large-scale population studies indicate that symptoms of AIWS are experienced quite frequently in the general population, with lifetime prevalence rates of up to 30% (5–7). Since the latter cases are typically fleeting and transient in nature, and seldom entail help-seeking behavior, they are referred to as “nonclinical cases.” Of the clinical cases, some 85% show involvement of only a single sensory modality. Of them, the majority are limited to a single symptom (e.g., only micropsia or only macrosomatognosia, etc.) (8). AIWS has many different etiologies. In clinical cases, encephalitis (notably due to Epstein-Barr virus infection) is the most common cause in children; in adults, it is migraine (4). Although prognosis and outcome are largely dependent on the underlying disorder and its amenability to treatment, the burden caused by AIWS may be substantial, even when its cause is fairly harmless, as in the context of fever or sporadic cannabis use. This is especially true when symptoms severely disrupt one's sensory perception, interfere with one's daily functioning, and/or evoke catastrophic thoughts (e.g., that the world has actually changed in an incomprehensible way or that symptoms may be indicative of dementia or schizophrenia). Since non-clinical cases are by definition self-limiting, and even in 50% of all clinical cases, reassurance would seem to suffice, AIWS is sometimes characterized as relatively harmless in nature (9). However, since research in this area is still in its infancy, and AIWS may well be severely underreported, it is too soon to draw any conclusions regarding its natural history and “typical” outcome. Moreover, we already know that AIWS can be a manifestation of severe and even lethal conditions, such as brain infarction or brain tumor (10, 11). We present here a case of AIWS caused by sporadic Creutzfeldt-Jakob disease (CJD). With it, we add to the burgeoning literature on AIWS, and seek to counter any premature conclusions that this syndrome can *a priori* be characterized as relatively “benign” in nature.

## Methods

The patient we describe was under treatment in our own clinical practice. Since he died within 2 months after his symptoms of AIWS had begun, we obtained written consent to publish from his family.

## Results–Case Report

In March 2018, a 68-year-old male reported to our neurology outpatient clinic with visual symptoms. He had a history of cataract surgery in 2004 and perception deafness beginning in 2006. A few weeks before we saw him, he had been referred to the ophthalmologist because of poor depth and size perception and a tight sensation around the head that had persisted for 1 month. He had no other (neurological) symptoms. Ophthalmological evaluation indicated an open-angle glaucoma with intraocular pressures of 39/27 mmHg ODS (reference 12–21 mmHg). His visual acuity was nearly normal (OD 0·8 dpt, OS 0·9 dpt), although the visual field examination revealed several mild defects (OD) that were not consistent with his symptoms. His eye pressure normalized with the use of bimatoprost and dorzolamide/timolol eye drops. However, over the course of the following weeks, the visual symptoms became more pronounced and peculiar. Upon referral to our outpatient clinic, he reported that moving objects were slowing down and speeding up “*like in a Charlie Chaplin movie*” (known as protracted duration and quick-motion phenomenon, respectively, i.e., variants of the group of time distortions). Sometimes he even failed to see any motion at all (akinetopsia). In addition, he perceived colors as exceptionally bright (hyperchromatopsia) and changing randomly, such as a red T-shirt turning green “*like fairground lights”* (chloropsia/dyschromatopsia). He also saw objects shrinking or swelling up to an unnatural size, “*as if he were looking into a funhouse mirror*” (micropsia and macropsia, zoom vision). In addition to these types of metamorphopsia, he developed paraesthesias in the hands and feet, gait instability and concentration and memory dysfunctions. He also had difficulty playing the jazz flute and saxophone, which we considered an ominous sign, given that he was a professional jazz musician. His speech became less fluent because he couldn't find the right words or failed to string them together to make sentences, and his partner noticed behavioral changes in the form of agitation and emotional lability.

**Figure 1:**
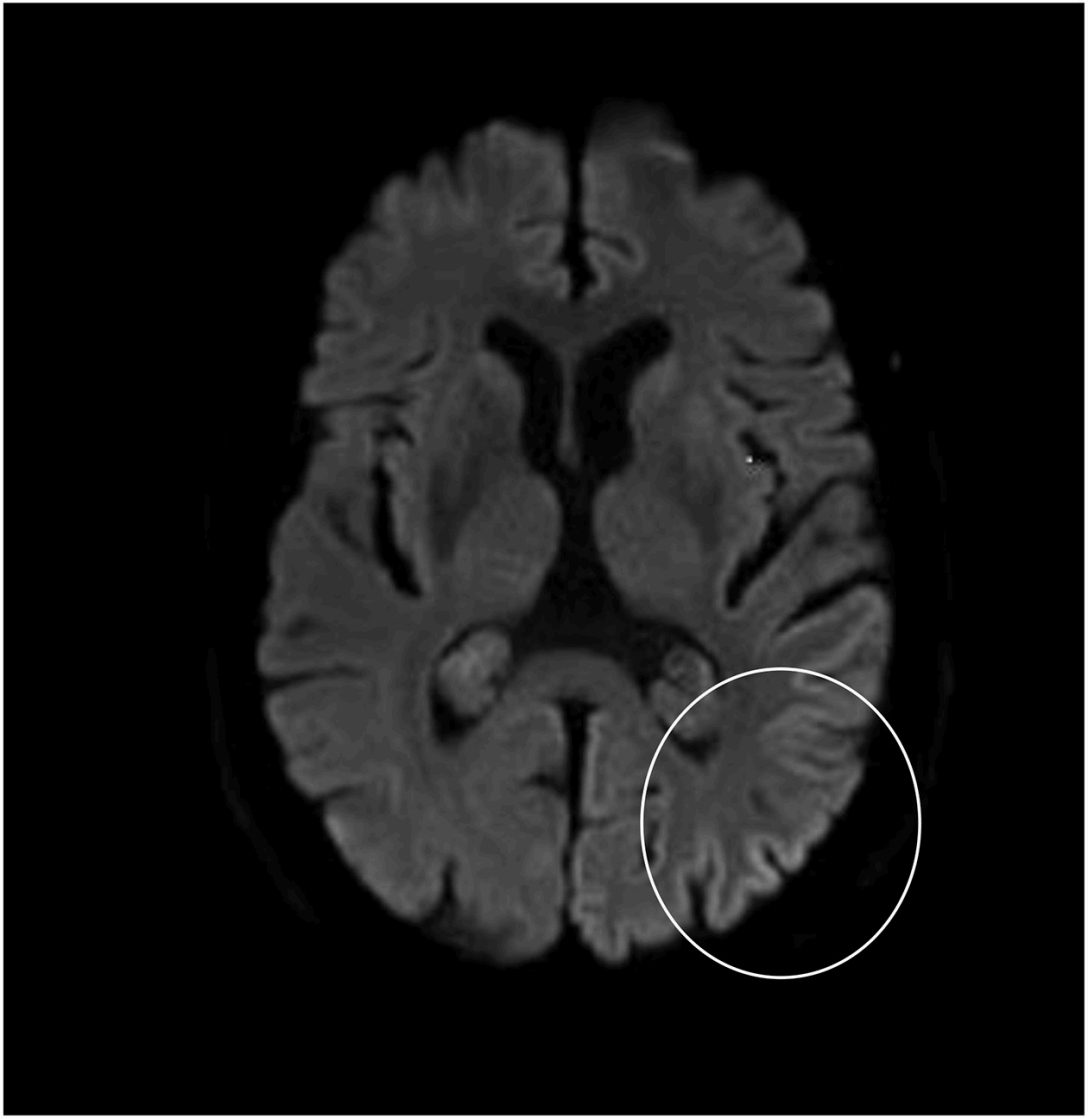
High signal intensity of the left parieto-occipital cortex on diffusion-weighted MRI (DWI; b = 1,000).

**Figure 2:**
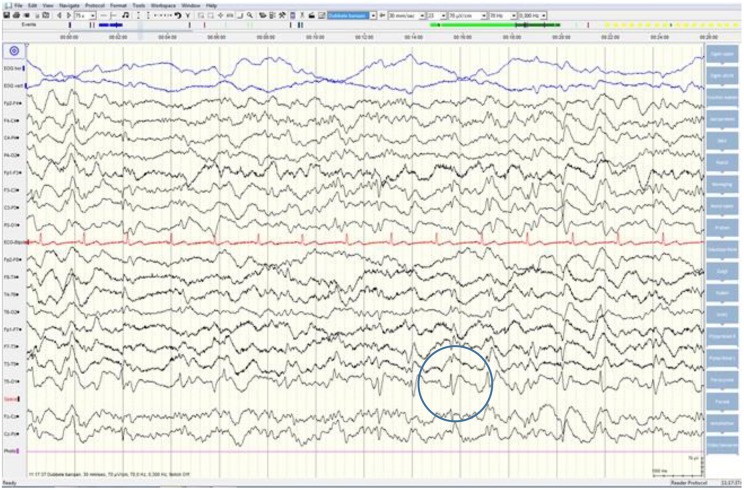
EEG showing periodic sharp-wave complexes over the left parieto-occipital region, suggestive of CJD.

Although neurological examination, routine blood testing, and a head MRI (including diffusion-weighted imaging, DWI) showed no abnormalities, our patient became increasingly forgetful and disoriented, and was subsequently admitted to our hospital. There we saw a previously eloquent man who now suffered from aphasia, ataxia, myoclonic jerks, and a rapidly progressive dementia. A second head MRI showed low apparent diffusion coefficient (ADC) values and increased DWI-signaling in the left parieto-occipital cortex, characteristic of a cortical ribbon sign (Figure 1), while the electroencephalogram showed periodic sharp-wave complexes (PSWC) over the same area (Figure 2). The MRI with presence of 14-3-3 proteins and elevated tau protein in the cerebrospinal fluid supported the diagnosis of probable sporadic Creutzfeldt-Jakob disease (CJD), notably the Heidenhain variant. We excluded other possible causes, including auto-immune disorders, infections, malignancies, toxic and metabolic encephalopathies, and other neurodegenerative diseases (notably posterior cortical atrophy and dementia with Lewy bodies). We did not carry out genetic testing because the family history was negative for neurodegenerative disease, CJD in particular. Unfortunately there were no therapeutic options. In conformity with his wishes, our patient was discharged home, where he died within 2 months after his visual symptoms had begun. We were unable to obtain autopsy consent.

## Discussion

What makes this case unique, is that our patient presented with a relatively large number of metamorphopsias (which, in the extant literature, is reported in only 15% of all clinical cases of AIWS) and that CJD was the most likely cause. Since a diagnosis of definite CJD can only be established post mortem by autopsy, it was unfortunate that we were unable to obtain autopsy consent. Still, MRI findings have been found to be positive in 83% of the cases of probable CJD (vs. in 98% of the cases of definite CJD) (12). Therefore, even though head MRI is not the gold standard, a diagnosis of CJD was still very likely in our patient, especially considering our additional neurological work-up, which failed to yield an alternative diagnosis that could explain the clinical picture, including its rapid decline and dramatic outcome.

### Alice in Wonderland Syndrome

As described by John Todd in 1955, AIWS is characterized by perceptual distortions reminiscent of the visual distortions, time distortions, bodily changes, derealization, and depersonalization experienced by Alice during her stay in Wonderland, as described in Lewis Carroll's classic children's book *Alice's Adventures in Wonderland* (1, 13). Todd, and before him, Lipmann (14), suggested that Carroll–whose real name was Charles Lutwidge Dodgson–had found inspiration for these peculiar phenomena in perceptual distortions he had experienced himself in the context of migraine. Although this hypothesis was followed by several historical treatises alleging that migraine was indeed the most likely source of Dodgson's experiences (15, 16), others have put forward the equally tantalizing hypothesis that, in his case, it was rather epilepsy that caused them (17) or perhaps substance abuse (18). Although we will probably never know for sure what made Dodgson experience these symptoms, a careful reconstruction of his medical history indicates that their most likely source was infectious disease, from which the author suffered on numerous occasions from childhood onwards, literally until the last day of his life (19). It is very likely that Dodgson had experienced these distortions himself (rather than having based his heroine's experiences on third-hand information), with a whopping 13 different symptoms described throughout the book, long before Todd had come up with the concept of AIWS.

To understand what makes AIWS stand out from other perceptual syndromes and disorders, we need to realize that distortions differ from hallucinations and illusions in that they are neither newly formed percepts of something that is not there (hallucination), nor actual objects or scenes mistakenly judged to be something else (illusion). Instead, distortions are percepts, experienced by a waking individual, which are based on appropriate stimuli from the outside world, of which a highly specific aspect is altered in a consistent manner [(20); for definitions, see Table 1]. Today, some 55 different types of perceptual distortion are known that may occur in the context of AIWS (4). The ones most frequently reported are micropsia (seeing things smaller than they are), macropsia (seeing things larger than they are), teleopsia (seeing things further away than they are), and dysmorphopsia (seeing straight lines as wavy). The provision that such aspects are altered *in a consistent manner*, means that the distortion applies to anything within the patient's visual range. Thus, people suffering from plagiopsia will typically see all things as slanted, in the same direction, and under the same degree, while those with prosopometamorphopsia will typically see one particular alteration in all the faces they perceive, such as heavy eyebrows *or* one eye that is markedly abducted to the nose *or* human faces changing into dragon faces (21, 22). The nature of these distortions may vary over time, but once present, all things perceived appear as if distorted in a similar manner.

**Table 1:** Definitions of hallucination, illusion, and distortion [after (20)].

Hallucination	A percept, experienced by a waking individual, in the absence of an appropriate stimulus from the outside world (e.g., seeing a cat that is not there, hearing a voice in the absence of sound waves)
Illusion	A percept, experienced by a waking individual, which is based on an appropriate stimulus from the outside world, and which is either misperceived or misinterpreted (e.g., taking a moving curtain for an intruder, hearing music in the monotonous drone of a computer fan)
Distortion	A percept, experienced by a waking individual, which is based on an appropriate stimulus from the outside world, of which, however, a highly specific aspect is altered in a consistent manner (e.g., seeing all straight lines as wavy, feeling one's head grow to an unnaturally large size)

The distinction between hallucination, illusion and distortion is not merely an academic issue. On the contrary, their phenomenological differences are thought to reflect differences in underlying mechanisms. Pathophysiologically, perceptual distortions are attributed to structural or functional lesions of discrete parts of the perceptual network, such as area V4 in hyperchromatopsia and V5 in akinetopsia (23). There are eight known groups of underlying etiology, comprising infectious diseases (encephalitis), central nervous system (CNS) lesions (stroke, brain tumor), peripheral nervous system (PNS) lesions (eye disease, middle-ear disease), paroxysmal neurologic disorders (epilepsy, migraine), psychiatric disorders (depression, schizophrenia), medications, illicit substances, and a miscellaneous group which includes hypnagogia and sensory deprivation (4). Thus, so-called “clinical cases” of AIWS always warrant diagnostic work-up, including neurological and psychiatric consultation, head MRI and EEG, and, when indicated, other auxiliary investigations (e.g., ophthalmologic examination, ENT consultation). Although evidence-based treatments are as yet to be developed, it is good clinical practice to aim therapy at the (probable) underlying cause (4, 19).

### Creutzfeldt-Jakob Disease

To our knowledge, AIWS has not been reported before in the context of CJD. With 1–1.5 cases per million population per year, CJD is an extremely rare disease that is fatal within a year in about 90% of those affected (24). The peak incidence is in the seventh decade, whereas younger (20–40 yrs) or older (>80 yrs) cases are much less common (25). CJD belongs to the transmissible spongiform encephalopathies or prion diseases. Prion diseases are characterized by the deposition of an abnormally misfolded isoform of the native prion protein, which is encoded by the prion gene (*PRNP*) on human chromosome 20. The mechanism for triggering this conformational change is unknown, but the accumulation of this abnormal prion protein leads to neuronal degeneration, astrocytic gliosis, and spongiform changes, accompanied by rapid cognitive decline, and ending in death. There are four known subtypes of CJD, comprising (i) sporadic CJD (sCJD), which is the most common form, accounting for approximately 85% of the cases; (ii) genetic CJD, which accounts for 10–15% of the cases and is associated with mutations of the prion gene (*PRNP*); (iii) the iatrogenic form of CJD, which accounts for approximately 1% of cases and is most frequently associated with prior treatment with human pituitary-derived hormones or human dura-mater grafts; and (iv) variant CJD (vCJD), which is a novel human prion disease found almost exclusively in the UK, and has been linked to the consumption of beef products contaminated with the causative agent of bovine spongiform encephalopathy (BSE) or “mad cow disease.” Moreover, based on the initial clinical presentation, six phenotypes have been identified, called cognitive, visual (Heidenhain variant), affective, classic, atactic (Oppenheimer-Brownell variant), and indeterminate (26). The Heidenhain variant was first described during the 1920s, and named after the German physiologist and histologist Rudolf Heidenhain (1834-1897). Representing some 20% of all cases of sporadic CJD (26–28), it is characterized by isolated visual symptoms at disease onset, such as visual field defects and loss of visual acuity, which are considered to be reflections of early involvement of occipital cortex (29). Since these visual defects may precede cognitive and motor symptoms by several weeks, it is not uncommon that CJD is not even suspected in such cases, and that patients are initially referred to an ophthalmologist.

### Conclusion

We conclude that the possibility of a Heidenhain variant of CJD, although extremely rare, needs to be entertained in the differential diagnosis of AIWS, especially in the presence of rapid cognitive decline. Moreover, we conclude that AIWS may be relatively harmless in the majority of cases, but that “clinical” cases always warrant a proper diagnostic work-up before this conclusion can be drawn.

### Limitations

Our knowledge of the numerous causes and manifestations of AIWS is still in its infancy. The syndrome was first conceptualized in 1955, but only the past few years have witnessed a steady rise in the number of publications on this disparate group of phenomena. Since the area is still very much in flux, our theoretical reflections on AIWS, although state-of-the-art, must be considered preliminary in nature. Secondly, the fact that we present here the first case report on AIWS in the context of CJD does not necessarily imply that our patient was the first person to ever suffer from a these two rare conditions. Notably, the Heidenhain variant of CJD can be expected to present more frequently with metamorphopsias. Therefore, our inability to find any prior case descriptions of symptoms of AIWS in the context of CJD may well be due to underreporting rather than to an actual rarity of the combination. A third and final limitation is that we were unable to obtain autopsy consent, which would have been necessary for making a diagnosis of definite CJD.

## Ethics Statement

Written consent to publish was obtained from the patient's family.

## Contribution to the Field Statement

Alice in Wonderland syndrome (AIWS) is a rare neurological disorder characterized by distortions of visual perception, the body image, and the experience of time. People may see things smaller than they are, feel their body alter in size or experience any of the syndrome's numerous other symptoms. Since there are also many known causes of AIWS, diagnosis requires a thorough neurological work-up. In children, the most common cause is brain inflammation; in adults, it is migraine. In the medical literature no more than 180 individual patients have been described, 50% of whom recovered. However, large-scale population studies indicate that fleeting, transient symptoms of AIWS are experienced by up to 30% of all healthy individuals. Accordingly, AIWS is generally considered fairly harmless in nature. We present here the first case description of an elderly male who suffered from AIWS due to Creutzfeldt-Jakob disease (CJD), an extremely rare neurodegenerative disorder. Our patient rapidly developed a state of dementia, and died within 2 months after his visual symptoms had begun. We conclude that AIWS is not always harmless, and that CJD, although extremely rare, must be on doctors' minds when the visual symptoms typical of AIWS are accompanied by rapid cognitive decline.

## Author Contributions

TN contributed to the conception and design of the work, and to the acquisition, analysis, and interpretation of data for the work, drafted and revised the work, gave final approval for the final version to be published, and agreed to be accountable for all aspects of the work in ensuring that questions related to the accuracy or integrity of any part of the work are appropriately investigated and resolved. BtM, and SvdW contributed to the conception and design of the work, and to the acquisition, analysis, and interpretation of data for the work, revised the work, gave final approval for the final version to be published, and agreed to be accountable for all aspects of the work in ensuring that questions related to the accuracy or integrity of any part of the work are appropriately investigated and resolved. JDB contributed to the conception and design of the work, and to the analysis and interpretation of data for the work, drafted and revised the work, gave final approval for the final version to be published, and agreed to be accountable for all aspects of the work in ensuring that questions related to the accuracy or integrity of any part of the work are appropriately investigated and resolved.

### Conflict of Interest Statement

The authors declare that the research was conducted in the absence of any commercial or financial relationships that could be construed as a potential conflict of interest.
